# Incidence and consequence of acute kidney injury in unselected emergency admissions to a large acute UK hospital trust

**DOI:** 10.1186/1471-2369-15-84

**Published:** 2014-05-29

**Authors:** Rachael Challiner, James P Ritchie, Catherine Fullwood, Paul Loughnan, Alastair J Hutchison

**Affiliations:** 1 Renal and Intensive Care Medicine, Manchester Royal Infirmary and Manchester Academic Health Science Centre, Central Manchester University Hospitals NHS Foundation Trust, Oxford Road, Manchester, M13 9WLUK; 2 Clinical Research Fellow, Manchester Academic Health Science Centre and University of Manchester, Manchester, UK; 3 Medical Statistician, Institute of Population Health, University of Manchester and Manchester Biomedical Research Centre, Central Manchester University Hospitals NHS Foundation Trust, Manchester Academic Health Sciences Centre, Manchester, M13 9WLUK; 4 Foundation Year 1, Royal Victoria Hospital, Belfast, UK; 5 Renal Medicine and (Specialist Medicine), Manchester Royal Infirmary and Manchester Academic Health Science Centre, Central Manchester University Hospitals NHS Foundation Trust, Oxford Road, Manchester, M13 9WLUK

**Keywords:** Acute kidney injury, Acute renal failure, AKI, Mortality, Length of stay, Critical care, Renal replacement therapy, Healthcare economics

## Abstract

**Background:**

AKI is common among hospital in-patients and places a huge financial burden on the UK National Health Service, causing increased length of hospital stay and use of critical care services, with increased requirement for complex interventions including dialysis. This may account for up to 0.6% of the total Health Service budget. To investigate the incidence and consequences of AKI, all unselected emergency admissions to a large acute UK single centre University Teaching Hospital over two separate 7 day periods were reviewed.

**Methods:**

A retrospective audit of 745 case records was undertaken (54.6% male) including laboratory data post-discharge or death, with classification of AKI by RIFLE, AKIN and AKIB criteria. Participants were included whether admitted via their general practitioners, the emergency department, or as tertiary specialty transfers. Outcome measures were presence or absence of AKI recorded using each of the three AKI criteria, length of hospital stay (LOS), admission to, and LOS in critical care, and mortality. The most severe grade of AKI only, at any time during the admission, was recorded to prevent double counting. Renal outcome was determined by requirement for renal replacement therapy (RRT), and whether those receiving RRT remained dialysis dependent or not.

**Results:**

AKI incidence was 25.4% overall. With approximately one third present on admission and two thirds developing post admission. The AKI group had LOS almost three times higher than the non AKI group (10 vs 4 days). Requirement for critical care beds was 8.1% in the AKI group compared to 1.7% in non AKI group. Overall mortality was 5.5%, with the AKI group at 11.4% versus 3.3% in the non AKI group.

**Conclusions:**

AKI in acute unselected hospital admissions is more common than existing literature suggests, affecting 25% of unselected admissions. In many this is relatively mild and may resolve spontaneously, but is associated with increased LOS, likelihood of admission to critical care, and risk of death. If targeted effective interventions can be developed it seems likely that substantial clinical benefits for the patient, as well as financial and structural benefits for the healthcare organisation may accrue.

## Background

In 2009 The National Confidential Enquiry into Patient Outcomes and Death (NCEPOD) report - “Adding Insult to Injury” - published a review of the care of patients who died in UK hospitals with a primary diagnosis of acute kidney injury [1]. The principal findings suggested that whilst organisational issues, such as delays in access to diagnostics and specialist care, contribute to the problems in managing Acute Kidney Injury (AKI), a more significant problem is failure of clinicians to recognise AKI at an early stage. This leads to delays in appropriate management and referral to specialist renal services, especially in patients who developed AKI post admission.

Acute kidney injury is a common but complex condition that occurs on all acute hospital sites and in all areas of the hospital, but which generally does not result in a requirement for dialysis treatment. It is accepted that AKI is associated with high individual morbidity and mortality, and places a huge financial burden on the UK National Health Service (NHS) in terms of increased length of hospital stay, plus increased requirement for complex interventions and critical care services. It is estimated that total yearly costs of AKI (including acute hospital admissions, critical care and RRT) was 0.4 - 0.6% of the total UK NHS budget (range between £434million and £620million in 2009–2010) [2-4] Added to this is the long-term cost of subsequent chronic kidney disease, including renal replacement therapy if end stage renal failure (ESRF) is reached [5].

No definitive studies have been undertaken within the UK to determine the precise prevalence of all stages of AKI. In the US the prevalence within hospitalised patients has been shown in the past to be 5-7% [6,7]. However these studies looked at ARF (Acute Renal Failure) rather than AKI as they pre-dated the 3 AKI classification systems we used. A more recent study from the US published this year, has shown AKI incidence of between 7.0-18.3% depending on which classification system was used [8]. The figure for patients within Intensive Care Units (ICU) is considerably higher, with rates of AKI of up to 85% and between 5-15% requiring RRT [9,10]. Data from the UK Intensive Care National Audit Research Centre (ICNARC) suggests that AKI accounts for nearly 10 percent of all ICU bed days [11]. AKI requiring RRT has been reported to account for 4.9% of all admissions to Intensive Care [12]. Comparison of these studies is made difficult by the varying definitions of AKI used in the literature. However, since 2004 more precise terminology and generally accepted definitions of AKI [13,14] have enabled healthcare professionals to define the disease more consistently, with a spectrum of injury severity ranging from milder self-limiting forms to more advanced injury possibly requiring RRT.

Despite these improvements in terminology, at the time of this study three definitions of AKI were in use (Table 1);

**Table 1:** Definitions used for the three scoring systems for AKI

**RIFLE**				**AKIN**		**AKIB**	
**Stage**	**Serum creatinine criteria**	**MDRD eGFR criteria**	**Anuria criteria**	**Stage**	**Aerum creatinine**	**Stage**	
**Risk**	>1.5× baseline over 7 days	>25% decrease over 7 days		**1**	> 1.5× baseline or >26 mmol/l increase over 48 hours	**1**	>26 mmol/l increase over 24 h or >44 mmol/l over 48 h
**Injury**	>2× baseline over 7 days	>50% decrease over 7 days		**2**	>2× baseline over 48 hours	**2**	>44 mmol/l increase over 24 h or >88 mmol/l over 48 h
**Failure**	>3× baseline over 7 days	>75% decrease over 7 days	anuria for >12 h	**3**	>3× baseline or 44 mmol/l increase if baseline > 300 mmol/l over 48 hours or any RRT or anuria for >12 h	**3**	>88 mmol/l increase over 24 h or >132 mmol/l over 48 h

Acute Dialysis Quality Initiative “*RIFLE*” criteria (risk, injury, failure, loss, End Stage Renal Disease), “*AKIN*” (Acute Kidney Injury Network), “*AKIB*” Acute Kidney Injury (Bonventre), *MDRD eGFR* (Modification of Diet in Renal Disease estimated Glomerular Filtration Rate.

1) The Acute Dialysis Quality Initiative “RIFLE” criteria [15] (risk, injury, failure, loss, ESRD), and.

2) “AKIN” (Acute Kidney Injury Network) criteria [16].

3) “AKI (Bonventre)” criteria, referred to in this manuscript as AKIB, which does not reference urine output.

For this reason data was collected which allowed analysis using all three definitions. This was to ensure the data would remain relevant for comparison in future studies when a clear definition is adopted, and also to allow comparison between the three different systems. The AKI guidelines published in 2012 by the international guideline group, Kidney Disease: Improving Global Outcomes (KDIGO) has now proposed a combination of RIFLE and AKIN, and this definition seems likely to be globally accepted [17,18]. Hopefully this will enable consistency in future comparisons of the incidence, outcomes and efficacy of therapeutic interventions for AKI.

Clinically AKI is characterised by a rapid reduction in kidney function which may result in a failure to maintain fluid, electrolyte and acid–base homoeostasis. However there is now increasing recognition that even a relatively small rise in serum creatinine in a variety of clinical settings is associated with worse outcomes and AKI has been demonstrated to be an independent risk factor for mortality [19-21]. Increasing severity of AKI is associated with a further increased risk of death, and this is independent of co-morbidity [21-23]. If early AKI can be rapidly identified and appropriate management promptly instituted it may be possible to reduce the associated increased mortality, although this is not certain.

### Aims

We wished to study the incidence and the consequences of acute kidney injury in all unselected emergency admissions to the large acute UK hospital centre in which our regional renal service is based.

## Methods

All emergency admissions to Manchester Royal Infirmary, over two separate seven day periods, were included whether admitted via their general practitioners, the emergency department, or as tertiary specialty transfers. This included all acute medical and surgical admissions via general practice or the emergency department, as well acute admissions via tertiary referrals to Cardiology, Haematology, Transplant medicine, Vascular and Cardiothoracic surgery and Ophthalmology. Maternity admissions were excluded because of the large numbers and low likelihood of AKI, as were patients whose admission was for less than 24 hours. Data from two separate weeks (September and February) were utilised because data collection over a continuous two week period was not possible for staffing reasons. Any readmissions were counted as a separate admission on each occasion whether readmitted in the 7 day study period or admitted in both weeks studied. A review of all case records of the 745 emergency admissions to the trust during these two separate week periods was conducted by RC, JR and PL. A small team was used for the case note review, all of whom have a clinical medical background in Nephrology and/or Intensive Care medicine. This allowed standardisation of methods. RC acted as the final adjudicator in cases of disagreement.

Using hospital biochemistry database and paper records, each set of notes was reviewed for evidence of AKI either on admission or at any time during the admission. Previous hospital biochemistry records were checked and the lowest of any prior serum creatinine or Modification of Diet in Renal Disease Glomerular Filtration Rate (MDRD eGFR) from the past 6 months was recorded as the ‘baseline’ renal function. Exact urine output volume measurements (ml/kg) were not possible to collect due to paucity and/or inaccuracy of data. If the admission serum creatinine or estimated MDRD eGFR were better than the result in the previous 6 month period then the better result was assumed to be the patient’s baseline renal function. If there was no recorded result within the past 6 months then the patient was assumed to have a ‘normal’ eGFR (75 mls/min) or serum creatinine of >60 umol/l if female or >80 umol/l if male, unless there was a definite history of renal impairment or chronic kidney disease (CKD). Each patient’s blood results throughout their entire hospital episode were reviewed. A computerised data collection form was utilised to allow data obtained from the hospital case records to be entered in a format that could be converted to an Excel spreadsheet for analysis.

The following information was obtained for each admission:

• Basic demographics including ethnicity

• Initial route of admission, and admitting specialty

• Weekday of admission was also noted as previous studies have suggested poorer outcomes for weekend admissions [24].

• Serum creatinine level and MDRD eGFR at admission.

• Appropriateness of repeat testing of blood urea nitrogen, creatinine and electrolytes was adjudicated. All patients with an MDRD eGFR < 60 mls/min were considered to require repeat testing (unless no change from a documented baseline creatinine or eGFR in the past 6 months). If patients had a single blood test only but eGFR > 60 mls/min then a clinical assessment was made by RC as to whether repeat testing was indicated [1].

• Presence or absence of AKI was recorded using each of the three AKI criteria - RIFLE, AKIN and AKIB to allow comparison between the three different systems, and also to ensure that the data remained relevant for comparison to future studies if a specific one of the 3 criteria was universally adopted. The most severe grade of AKI only, at any time during the admission, was recorded in order to prevent double counting. The baseline renal function was taken as either the lowest of their admission or previous 6 month serum creatinine or eGFR if a baseline result was available. If there was no baseline then a ‘normal’ eGFR of >75 mls/min was assumed.

• Time of development of AKI – either present on admission (pre-admission), or developing during the hospital stay (post admission). If the creatinine or eGFR were abnormal on admission and no baseline was available (and hence assumed to be a pre admission AKI) then for the purposes of this study and scoring of the severity then the time in rise of creatinine or fall in eGFR was assumed to be within 7 days when using RIFLE and 48 hours using AKIN.

• Presence of oliguria (defined as urine output < 400 mls/day) and anuria was recorded.

• Documentation of urinalysis being performed. The complete nursing, medical notes and observations charts were reviewed and if there was no documentation of the result then it was assumed not to have been performed.

• Ultrasound scans of kidneys within 24 hours of development of AKI.

• Presence or absence of diabetes mellitus, hypertension, sepsis, known CKD and administration of contrast, all types of diuretics, Angiotensin converting enzyme inhibitors (ACEi) or Angiotensin receptor blockers (ARBs) and Non-steroidal anti-inflammatories (NSAIDS) as well as age > 65 and age > 80 was collected. Sepsis was defined as presence of at least two of the following indicators - white cell count <4×10^9^/L or >12×10^9^/L, C reactive protein (CRP) >50 mg/L and pyrexia (defined as temperature >38.0 degrees centigrade).

• Outcome. We recorded length of hospital stay, admission to, and length of stay in, critical care bed, and critical care and hospital mortality. The renal outcome was determined by the requirement for renal replacement, and whether those receiving RRT remained dialysis dependant or not.

The governance framework regarding the conduct and publication of audit projects in our centre is that all such projects are registered in the audit register of the trust/centre. Therefore ethical committee (national or regional) approval was not required and so not sought for this study. As it was an anonymous audit and case review of notes and biochemistry results written informed consent was not required for the same reason.

### Statistical analysis

Summary descriptive statistics were used to explore the demographics and admission characteristics of the sample. Relationships between risk factors and development of an AKI were explored via linear and stepwise multiple logistic regression. The continuous outcome, hospital length of stay was expressed by medians and compared between AKI groups with a Mann–Whitney test, whilst the Kruskal-Wallis test was used to assess the correlation between severity of injury and length of stay. Odds ratios were used to compare dichotomised the outcomes, admission to critical care and hospital mortality for AKI severity. All statistical analysis was conducted in R v2.15.2 with libraries gmodels, car, MASS, Epi, Hmisc, and epitools [25-30].

## Results

### Demographics

The total population comprised 745 patients, 54.6% male, with 385 in the initial week, and 360 in the second week. 6 patients whose admission was less than 24 hours were not included, and no patient was admitted more than once. There were no statistical differences between the weeks in any demographic characteristics.

The ethnic mix of the admissions reflected the population of a large UK inner city hospital. 78.9% were documented as White, 6.7% Black, 12.6% Asian, 1.1% Chinese, 0.4% Mixed race and 0.3% Hispanic.

86.2% (N = 642) of admissions to the hospital were via the emergency department, whilst the initial route of admission for the remaining patients was 7.4% Medical Admissions Unit, 1.1% Coronary Care Unit, 1.5% Surgical Assessment Unit and 3.9% other (via outpatient clinic or inter hospital transfer).

The distribution of the admitting specialty was 63.0% general medicine, 27.1% general surgery (includes urology/orthopaedics), 3.1% other/tertiary medical specialties (including haematology and renal) and 6.8% other/tertiary surgical specialties (renal transplant, cardiothoracic surgery, vascular surgery).

The admissions were relatively equally split across the days of the week (range 11.4% - 17.2%), although there were slightly higher admissions towards the end of the week, with fewer on Sunday and Monday.

### Incidence and investigation of AKI

95.7% of the 745 patients admitted had their serum creatinine and estimated Glomerular Filtration Rate (MDRD eGFR) checked on admission. 71.7% had at least two serum creatinine results, but a further 5.9% who were considered by the clinicians reviewing the patients notes to have required a repeat blood test, did not.

The baseline serum creatinine and eGFR were immediately available in 342 (45.9%) of the 745 total population and 333 (45.7%) of the group when the 17 dialysis patients were excluded. In these patients the prior blood result would have been available to the admitting clinician when reviewing the patient’s admission bloods at time of initial presentation.

7.0% of the total patients admitted were known to the renal service. 17 (2.3%) admissions were maintenance dialysis patients (either haemodialysis or peritoneal dialysis) and these were excluded from the analysis since they had no effective renal function. 22 (2.9%) of the total admissions were known to have pre-existing CKD 4 or 5, and were under renal follow-up but were included in the study since such patients may still present with, or develop AKI.

Of 728 patients included in the analysis, 185 (25.4%) were judged to have AKI at some time during their admission, using one of the three different criteria (Table 2). Each level of AKI is exclusive, so once a patient was allocated to a higher stage, they were excluded from all lower ones. The grade of severity of their AKI, and the differences using the three separate scoring systems are also shown in Table 2. RIFLE and AKIN both give a similar incidence at the more severe end of the spectrum with RIFLE-F (2.7%) and AKIN stage 3 (2.9%) incidence, and the same total incidence, 23.6%. AKIB gives a lower overall incidence at 21.4% and the split across the 3 stages differs from the other two scoring systems.

**Table 2:** **Incidence of AKI judged by each of three scoring criteria** (**full cohort**)

**RIFLE**		**AKIN**		**AKIB**	
**Stage**		**Stage**		**Stage**	
**Risk**	14.3% (104)	**1**	16.1% (117)	**1**	9.1% (66)
**Injury**	6.6% (48)	**2**	4.7% (34)	**2**	8.2% (60)
**Failure**	2.8% (20)	**3**	2.9% (21)	**3**	4.1% (30)
**Total**	23.6% (172)	**Total**	23.6% (172)	**Total**	21.4% (156)

Acute Dialysis Quality Initiative “*RIFLE*” criteria (risk, injury, failure, loss, End Stage Renal Disease), “*AKIN*” (Acute Kidney Injury Network), “*AKIB*” Acute Kidney Injury (Bonventre), *MDRD eGFR* (Modification of Diet in Renal Disease estimated Glomerular Filtration Rate.

Seven patients (0.9% of total population) required acute renal replacement therapy (RRT). This equates to approximately one third of the stage 3 AKI groups, (30.0% of RIFLE-F and 33.3% of AKIN stage 3 and 16.7% of AKIB stage 3). 2.3% of the admitted patients were on chronic RRT and so altogether more than 3% of the admitted patients needed dialysis.

Of all patients developing AKI, 106 had AKI present on admission (pre-admission AKI) and 79 patients had AKI which developed during their hospital stay.

Only 103 patients, 14.1% of the study population (excluding the 17 ESRF patients) underwent urinalysis, despite the NCEPOD recommendation that this is advisable for all emergency admissions.

Assessment of compliance with the UK Renal Association’s guideline that all AKI stage 3 patients should have a renal ultrasound within 24 hours of their diagnosis was difficult. 106 (14.2%) of the total admissions underwent renal ultrasonography, but the majority of these appeared to be as part of other investigations (such as for abdominal pain) rather than specifically requested due to renal impairment. Of the stage 3 AKI group RIFLE 9(45%), AKIN 10(47.6%), AKIB 12(40%) had an ultrasound of their renal tract during the hospital stay.

### Risk factors

Each patient was assessed for multiple potential risk factors associated with AKI. On univariate analysis the presence of diabetes mellitus, hypertension, known CKD, sepsis, diuretics, ACEi or ARBs and age > 65 were all associated with an increased odds ratio for development of AKI, with p-values all less than 0.05. For hypertension, known CKD, sepsis, diuretic use and age > 65 the p values were all <0.0001. Only NSAID usage did not show increased odds of developing AKI.

In the multivariate logistic regression fewer predictors remained significant in the full model once adjusted for the others, but known CKD, sepsis and diuretic usage were all significant (Table 3). These were all identified as important, alongside age over 65 by stepwise regression. When age is used in the model as a continuous predictor then similar results are found.

**Table 3:** **Analysis of risk factors for Acute Kidney Injury using multivariate logistical regression** (**full model**)

	**Odds ratio**	**C.I. lower**	**C.I. upper**	**P-value**
**Known CKD**	4.020	2.414	6.693	<0.001
**NSAID**	1.009	0.486	2.097	0.980
**Contrast**	0.937	0.562	1.565	0.804
**ACEi**	1.195	0.746	1.914	0.458
**Diuretic**	1.872	1.173	2.986	0.008
**Diabetic**	1.025	0.638	1.647	0.917
**Sepsis**	7.239	4.589	11.418	<0.001
**Hypertension**	1.438	0.904	2.288	0.125
**Age**	1.020	1.008	1.032	0.001
**Week**	0.770	0.511	1.161	0.212

Chronic Kidney Disease (*CKD*) as Modification of Diet in Renal Disease estimated Glomerular Filtration Rate (MDRD eGFR < 60 mls/min, Non-steroidal anti-inflammatory drugs (*NSAID*), Angiotensin converting enzyme inhibitors (*ACEi*), Confidence interval (*C.I*).

As the total population was a combination of two separate week’s admissions data, week was included in the multivariate analysis as a predictor. It was found not to be important demonstrating that there were no intrinsic differences between our two sample weeks.

### Outcomes

**Figure 1:**
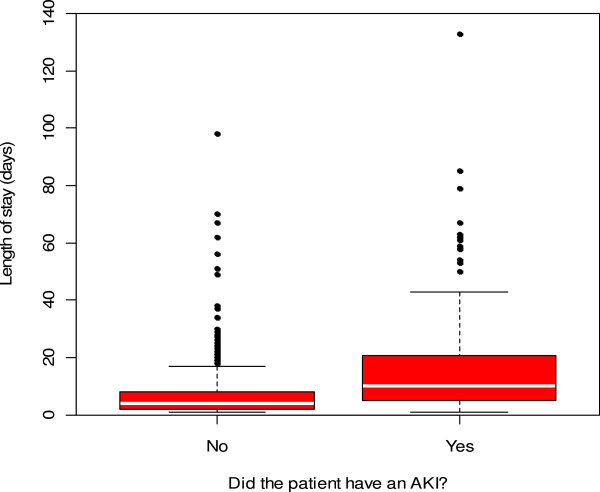
**Difference in hospital length of stay in patients with and without an Acute Kidney Injury (AKI) episode (using AKI as identified by any of the 3 scoring systems).**

**Figure 2:**
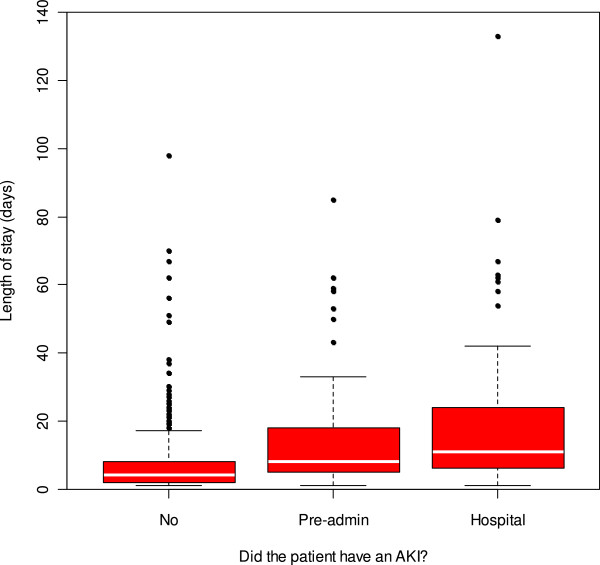
Differences in hospital length of stay depending on timing of episode of Acute Kidney Injury.

**Figure 3:**
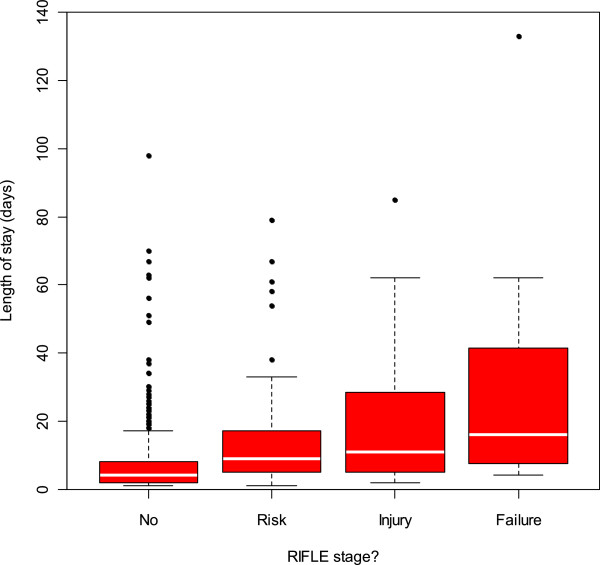
**Hospital length of stay increases with severity of Acute Kidney Injury (using the RIFLE definitions).**

The length of hospital stay (LOS) tended to be greater in patients with AKI (see Figure 1). The median LOS in the AKI group was 10.0 days versus 4.0 days in the patients without AKI which was found to be statistically significant (p < 0.0001) via a Mann–Whitney test. There was no significant difference in LOS between those patients who were admitted with AKI (median = 8.0 days), and those who developed AKI after admission (median = 11.0 days) (Mann–Whitney, p = 0.05, see Figure 2).The severity of the injury correlates with length of hospital stay, use of critical care beds and risk of death. These findings hold true whichever of the three scoring systems is used to define AKI. Figure 3 illustrates LOS under the RIFLE criteria, which clearly shows increasing length of stay with degree of injury (Kruskal-Wallis p < 0.0001, spearmans correlation = 0.37).

3.5% (26 patients) of the cohort had a critical care admission during the hospital stay. Of those patients with AKI, 8.1% were admitted to critical care, compared to only 1.7% of those without AKI. The mortality of the 26 patients admitted to critical care was considerably higher (23.1%) than the overall hospital mortality, which for the total emergency admissions population was 5.4%. Two of these were ESRF dialysis patients. Of the critical care survivors, 10.5% (2 patients) remained dialysis dependent. Patients who developed AKI in hospital were no more likely to require admission to critical care (9.0%) than those developing AKI pre admission (7.5%), however this was not significant (odds 1.2, p = 0.74).

In each scoring system ‘risk’ or stage 1 AKI does not appear to increase the likelihood of a critical care admission. However, the proportion admitted to Critical Care almost doubles between ‘injury’ and ‘failure’ (RIFLE criteria) and stages 2 and 3 (using AKIN criteria) although the numbers involved are small with wide binomial confidence intervals (see Table 4). Approximately 20% of the patients with AKI require critical care support, although not all needed or received renal replacement therapy.

**Table 4:** **Critical care usage increase with severity of AKI using three scoring systems with patient numbers** (**percentage of individuals in each category**) (**Binomial 95**% **confidence intervals**)

**RIFLE**		**AKIN**		**AKIB**	
**Stage**	**Patients admitted to critical care**	**Stage**	**Patients admitted to critical care**	**Stage**	**Patients admitted to critical care**
**No AKI**	12 (2.2%)	**No AKI**	10 (1.8%)	**No AKI**	12 (2.1%)
	(1.1 to 3.7)		(0.9 to 3.3)		(1.1 to 3.6)
**Risk**	3 (2.9%)	**1**	5 (4.3%)	**1**	1 (1.5%)
	(0.6 to 8.2)		(1.4 to 9.7)		(0.0 to 8.2)
**Injury**	5 (10.4%)	**2**	4 (11.8%)	**2**	7 (11.7%)
	(3.5 to 22.7)		(3.3 to 27.5)		(4.8 to 22.6)
**Failure**	4 (20.0%)	**3**	5 (23.8%)	**3**	4 (13.3%)
	(5.7 to 43.7)		(8.2 to 47.2)		(3.8 to 30.7)

Acute Dialysis Quality Initiative “*RIFLE*” criteria (risk, injury, failure, loss, End Stage Renal Disease), “*AKIN*” (Acute Kidney Injury Network), “*AKIB*” Acute Kidney Injury (Bonventre), *MDRD eGFR* (Modification of Diet in Renal Disease estimated Glomerular Filtration Rate.

The hospital mortality for the AKI group was 11.4% (7.2 to 16.9) compared to only 3.3% (2.0 to 5.2) of the admissions who did not have AKI (see Table 5). The odds of death in the AKI group compared to those without AKI was 3.7, which was highly significant (p < 0.0001), (95% C.I. odds 1.94-7.30). The hospital mortality is similar for pre admission AKI (11.3%), or post admission AKI (11.4%).

**Table 5:** **Hospital mortality increases with severity of AKI using three scoring systems with patient numbers** (**percentage of individuals in each category**) (**Binomial 95**% **Confidence Intervals**)

**RIFLE**		**AKIN**		**AKIB**	
**Stage**	**Hospital mortality**	**Stage**	**Hospital mortality**	**Stage**	**Hospital mortality**
**No AKI**	20 (3.6%)	**No AKI**	19 (3.4%)	**No AKI**	19 (3.3%)
	(2.2 to 5.5)		(2.1 to 5.3)		(2.0 to 5.1)
**Risk**	7 (6.8%)	**1**	12 (10.3%)	**1**	5 (7.6%)
	(2.8 to 13.5)		(5.5 to 17.4)		(2.5 to 16.8)
**Injury**	7 (14.6%)	**2**	5 (14.7%)	**2**	10 (16.9%)
	(6.1 to 27.8)		(5.0 to 31.1)		(8.4 to 29.0)
**Failure**	5 (25.0%)	**3**	3 (14.3%)	**3**	5 (16.7%)
	(8.7 to 49.1)		(3.0 to 36.3)		(5.6 to 34.7)

Acute Dialysis Quality Initiative “*RIFLE*” criteria (risk, injury, failure, loss, End Stage Renal Disease), “*AKIN*” (Acute Kidney Injury Network), “*AKIB*” Acute Kidney Injury (Bonventre), *MDRD eGFR* (Modification of Diet in Renal Disease estimated Glomerular Filtration Rate.

Whilst there is noticeable overlap, there is a definite increase in mortality with increasing severity of AKI. Patients with stage 1 AKI (Rifle risk) have an odds ratio of death of 2.0 (0.7 to 4.6, midp.exact p = 0.158), stage 2 (RIFLE-I equivalent) an odds ratio of 4.6, (1.7 to 11.2, p = 0.004) and increasing to 9.0 (2.7 to 26.3, p = 0.001) for stage 3 (RIFLE-F).

### Sub-analysis of patients with known prior information of renal function

339 patients are included in the sub-analysis, 129 (38.1%) were judged to have AKI at some time during their admission, using one of the three different criteria (Table 6). As in the full cohort RIFLE and AKIN both give a similar incidence at the more severe end of the spectrum with RIFLE-F (4.1%) and AKIN stage 3 (5.0%) incidence, and similar total incidence, 35.1% and 35.4% respectively. AKIB gives a lower overall incidence at 32.4% and again the split seen across the 3 stages differs from the other two scoring systems.

**Table 6:** **Incidence of AKI** (**in sub group of patients with known baseline renal function**) **judged by each of three scoring criteria**

**RIFLE**		**AKIN**		**AKIB**	
**Stage**		**Stage**		**Stage**	
**Risk**	21.5% (73)	**1**	23.9% (81)	**1**	14.5% (49)
**Injury**	9.4% (32)	**2**	6.5% (22)	**2**	11.5% (39)
**Failure**	4.1% (14)	**3**	5.0% (17)	**3**	6.5% (22)
**Total**	35.1% (119)	**Total**	35.4% (120)	**Total**	32.4% (110)

Acute Dialysis Quality Initiative “*RIFLE*” criteria (risk, injury, failure, loss, End Stage Renal Disease), “*AKIN*” (Acute Kidney Injury Network), “*AKIB*” Acute Kidney Injury (Bonventre), *MDRD eGFR* (Modification of Diet in Renal Disease estimated Glomerular Filtration Rate.

Six patients (1.8% of sub-analysis) required renal replacement therapy (RRT). This equates to approximately one third of the stage 3 AKI groups, (35.7% of RIFLE-F and 35.3% of AKIN stage 3 and 22.7% of AKIB stage 3).

Of all patients with known baseline renal function developing AKI, 76 had AKI present on admission (pre-admission AKI) and 53 patients had AKI which developed during their hospital stay.

### Sub group risk factors

As with the total cohort, on univariate analysis the presence of diabetes mellitus, hypertension, known CKD, sepsis, diuretics and age > 65 were all associated with an increased odds ratio for development of AKI, with p-values all less than 0.05. For known CKD, sepsis and diuretic use the p values were all <0.0001 in the sub group.

In the multivariate logistic regression fewer predictors remained significant once adjusted for the others, but known CKD, sepsis and diuretic use were all significant. These are also identified as most important by stepwise regression. When age is used in the model as a continuous predictor then the same results are found.

As the total population was a combination of two separate week’s admissions data, week was included in the multivariate analysis as a predictor. It was found not to be important demonstrating that there were no intrinsic differences between our two sample weeks.

### Sub group outcomes

As with the total cohort, in the sub group the length of hospital stay (LOS) tended to be greater in patients with AKI. The median LOS in the AKI group was 9.0 days versus 5.0 days in the patients without AKI which was found to be statistically significant (p < 0.0001) via a Mann–Whitney test. There was also significant difference in LOS between those patients who were admitted with AKI (median = 8.0 days), and those who developed AKI after admission (median = 14.0 days) (Mann–Whitney, p = 0.005).The severity of the injury correlates with length of hospital stay (see Figure 3), use of critical care beds and risk of death. These findings hold true whichever of the three scoring systems is used to define AKI. For LOS under the RIFLE criteria, we see increasing length of stay with degree of injury (Kruskal-Wallis p < 0.0001, spearmans correlation = 0.42).

3.5% (12 patients) of the subgroup had a critical care admission during the hospital stay. Of those patients with AKI, 6.2% were admitted to critical care, compared to only 1.9% of those without AKI. The mortality of the 12 patients admitted to critical care was considerably higher (23.1%) than the overall hospital mortality, which for the total emergency admissions population was 5.4%. Of the critical care survivors, 10.0% (1 patient) remained dialysis dependent. Patients who developed AKI in hospital were less likely to require critical care (5.7%) than those developing AKI pre admission (6.6%), however this was not significant (odds 0.9, p = 0.86).

The hospital mortality for the AKI group was 7.8% (3.8 to 13.8) compared to only 4.3% (2.0 to 8.0) of the admissions who did not have AKI. The odds of death in the AKI group compared to those without AKI was 1.9, which was non-significant (p = 0.193), (95% C.I. odds 0.72-4.90). The hospital mortality is similar for pre admission AKI (8.6%), or post admission AKI (7.4%).

Whilst there is noticeable overlap, there is a definite increase in mortality with increasing severity of AKI. Patients with stage 1 AKI (Rifle risk) have an odds ratio of death of 0.9 (0.2 to 3.2, midp.exact p = 0.917), stage 2 (RIFLE-I equivalent) an odds ratio of 3.0, (0.8 to 10.0, p = 0.107) and increasing to 3.6 (0.5 to 16.4, p = 0.183) for stage 3 (RIFLE-F).

## Discussion

Our study demonstrates that AKI occurs in almost a quarter of emergency admissions in a large single centre university teaching hospital. This is even higher (38.1%) in the subgroup of patients where true baseline renal function was known at the time of admission. However the incidence of AKI varies depending on which scoring system was used. The differences between the less severe stages when using RIFLE or AKIN are small, and this is consistent with what has been seen in previous validation studies for both systems. The time criteria used in the definition for each system, with RIFLE using a change in serum creatinine or eGFR over 7 days as opposed to 48 hours is the most likely explanation for this.

The AKIB system, which does not include urine output, gives a lower incidence of total AKI at 20.8%. This suggests that urine output may be more sensitive than serum creatinine as an early indicator of renal dysfunction (although probably much more difficult to assess accurately).

Whilst the overall incidence of AKI is higher at 38.1% in the group when baseline renal function was known compared to 25.4% in the whole group the breakdown across RIFLE or AKI stages 1/2/3 was almost identical. RIFLE R 60.5% (whole cohort) 61.3% (known baseline cohort), RIFLE I 27.9% (whole cohort) 26.9% (known baseline cohort), RIFLE F 11.6% (whole cohort) 11.8% (known baseline cohort). This is consistent with what has been seen in the Zeng US study with a split of AKI 1 70.9%, AKI 2 17.2%, AKI 3 12.0%.

Irrespective of the definition used, in both the total cohort and sub group analysis, we have shown AKI has a significant impact on length of hospital stay, use of critical care beds and mortality, even at the lower end of the severity of injury, which has also been seen in large studies in Italy and the US [8,20]. In our study AKI is associated with increased length of hospital stay, increased likelihood of admission to critical care (odds = 5.2, 2.3 to 12.7), and increased risk of death (odds = 3.7, 1.9 to 7.3). The presence of AKI and its severity correlated positively with both length of stay and hospital mortality. Median length of stay in the total cohort more than doubles from 4.0 days without AKI, up to 10.0 days with AKI (subgroup 5.0 days without AKI, up to 9.0 days with AKI). Although the more severe AKI episodes were small in number, so limiting the statistical power, the odds ratio of death increases from 2.0 with stage 1 to 9.0 for stage 3 (RIFLE-F). The figures in our study are comparable to other studies which have shown odds ratio of 2.0 for death in stage 1 and 10.1 in stage 3 [8].

Previous reports demonstrate that patients receiving RRT make up a small proportion of those reaching RIFLE-F in an ICU, yet the hospital mortality rate is greater than five times higher than that of the same ICU population without AKI [20,31-33]. In our study we also find that hospital mortality in critical care patients is nearly five times higher in patients with AKI (of any stage) compared with those patients that do not develop an AKI (8.1 vs 1.7% respectively). In the past this has been attributed to AKI simply being an indicator of illness severity, but this is now being challenged [34]. The increased morbidity and mortality seen with increasing severity of AKI is associated with an increased risk of “non-renal” complications such as bleeding and sepsis, but AKI may also influence remote organ function [35]. In the total cohort and subgroup, approximately one third of patients reaching RIFLE F required RRT. This has significant cost and resource implications, and if chronic dialysis patients’ admissions are included this equates to 3% of our total admissions.

Although the weeks studied were not consecutive we found no statistical differences between the patients in each week, and the variation in admission profile in the overall number of admissions, demographics, gender, age which could have affected the incidence of AKI were not different. Although the study was conducted in a single academic centre, only 9% of the admitted patients were under tertiary specialties and only 7% of patients were already known to the renal services, we feel our data is generally applicable to all but the smallest secondary care centres. Although the study is not as numerically large as some others, its major strength is that all admissions were captured and each case scrutinised by a nephrologist making it unlikely that any cases were missed, as can occur when relying only on electronic records as other larger studies have done [8,20,32]. Furthermore our study population included very few patients (4.3%) who did not have any serum creatinine values compared to nearly 24% in Zeng’s study [8].

A limitation which affects most studies in AKI, including ours, is the absence of a baseline creatinine for many patients hindering accurate assessment of baseline renal function. In our study 45% of patients had a known baseline, and so an estimated baseline was required in the remainder. In these cases we assumed a baseline eGFR of 75 mls/min per 1.73 m [2] as recommended by the Acute Dialysis Quality Initiative, and used a serum creatinine of 60 umol/L for females and 80 umol/L for males (as the midpoint of our laboratory normal range). Recently published data from Siew et al. suggests that using many multiple imputation methods to calculate baseline function improve AKI misclassification but this has yet to be commonly accepted [36]. Other authors have encountered similar difficulties so that Hoste et al. used a ‘back calculated creatinine’ via MDRD using an assumed eGFR of 75 mls/min in approximately 50% of their cohort and Zeng et al. used an estimated baseline in 24.6% of their cohort. Back calculation via eGFR has its own problems since it is well recognised that estimated GFR is wildly inaccurate when serum creatinine is normal or near normal (+30% from true GFR), and serum creatinine needs to have been stable for at least 4 days before it approximates to true GFR.

Using RIFLE our AKI incidence of 23.6% is higher than that previously reported. The recent study by Zeng et al., demonstrated an incidence of 16.1% (using RIFLE) and Uchino 18.0%, both studies using a cohort including all hospitalisations. Our sub group analysis using the population with a known baseline suggested an even higher incidence, between 32.4 – 35.4% depending on the AKI definition used. Interestingly, the Zeng study also found an increased incidence when analysing only the sub-group with a known baseline creatinine value. Their variation from 18 to 33% depending on the definition of AKI used, and also depending on how estimation of unknown baseline renal function was calculated, gives an incidence comparable to our results, and higher than previous studies. The higher overall incidence of AKI at 67.4% in the Hoste study most likely reflects that it was studying a very different group of patients using an ICU population only [32].

The potential risk factors assessed in the study are all commonly found co-morbidities in any hospital population. The significant effects of age, sepsis, diuretic use and pre-existing CKD highlight the importance of these factors in the development of AKI - aspects which may be under-recognized by clinicians. Educating clinicians to identify ‘at risk’ patients, both at the time of admission and also prior to this, will become increasingly important as we deal with an ageing population and its associated increased incidence of CKD. ‘Classical’ risk factors for AKI such as angiotensin blockade and radio-contrast use seem to play a smaller role than anticipated as risk factors for development of AKI in this and other studies. This may be due to already increased awareness of these modifiable factors. NSAIDs were not shown to be a significant risk factor despite being a well-recognised risk factor. The increased awareness of their association with renal dysfunction in an acute and chronic setting means has reduced their use in the elderly, CKD and heart failure patients – all groups which are at higher risk of AKI.

In summary, this study demonstrates that AKI is very common in acute unselected hospital admissions. Risk factors present at the time of admission suggest that it may be predictable, and therefore in some cases avoidable. If so, we calculate that a modest reduction of 10% in the incidence of AKI could save around 3,000 bed days per annum in similarly sized acute hospitals with 900–1000 beds. Whilst this has significant financial implications, of even greater importance is the benefit to the individual patient in terms of reduced morbidity, length of stay, long term renal outcome, and in some cases likelihood of death. To achieve such a reduction in the incidence of AKI would require a reliable method of early identification of the ‘at risk’ patient, and in particular those in whom AKI may be avoidable.

## Conclusion

AKI in acute unselected admissions to a large acute hospital is common, affecting around 25% of patients. In many cases this is relatively mild and may resolve spontaneously, however it is associated with increased length of hospital stay, increased likelihood of admission to critical care, and increased risk of death. Despite the publication of the NCEPOD report into AKI, routine investigations and opportunities to intervene appropriately may still be being overlooked. Risk factors for AKI can be identified and may be of use in alerting clinicians to ‘at risk’ patients who could then be monitored more closely. If targeted effective interventions can be developed it seems likely that substantial clinical benefits for the patient, as well as financial and structural benefits for the healthcare organisation may accrue.

## Competing interests

All authors declare that they have no competing interests.

## Authors’ contributions

Role of authors. RC was responsible entirely for the conception and design of the study. RC designed data collection tools, collected and monitored data collection for the whole trial, cleaned, analysed and interpreted the data and drafted then revised the paper and had final approval of the version to be published. JR and PL were involved in data collection. CF provided the statistical analysis and critical revision of the article for statistical validity. AJH analysed and interpreted the data, revised the paper for important intellectual content and had final approval of the version to be published. All authors read and approved the final manuscript.

## Authors’ information

The Corresponding Author has the right to grant on behalf of all authors and does grant on behalf of all authors, *a worldwide licence* to the Publishers and its licensees in perpetuity, in all forms, formats and media (whether known now or created in the future), to i) publish, reproduce, distribute, display and store the Contribution, ii) translate the Contribution into other languages, create adaptations, reprints, include within collections and create summaries, extracts and/or, abstracts of the Contribution, iii) create any other derivative work (s) based on the Contribution, iv) to exploit all subsidiary rights in the Contribution, v) the inclusion of electronic links from the Contribution to third party material where-ever it may be located; and, vi) licence any third party to do any or all of the above.”

## Pre-publication history

The pre-publication history for this paper can be accessed here:

http://www.biomedcentral.com/1471-2369/15/84/prepub
